# Academic Detailing Interventions and Evidence-Based Prescribing: A Systematic Review

**DOI:** 10.1001/jamanetworkopen.2024.53684

**Published:** 2025-01-08

**Authors:** Benjamin N. Rome, Ellen Dancel, Alexander Chaitoff, Dominick Trombetta, Shuvro Roy, Paul Fanikos, Jayda Germain, Jerry Avorn

**Affiliations:** 1 Division of Pharmacoepidemiology and Pharmacoeconomics, Department of Medicine, Brigham and Women’s Hospital, Boston, Massachusetts; 2 Harvard Medical School, Boston, Massachusetts; 3 Alosa Health, Boston, Massachusetts; 4 Department of Internal Medicine, Michigan Medicine, Ann Arbor, Michigan; 5 Department of Pharmacy Practice, Wilkes University, Wilkes-Barre, Pennsylvania; 6 Department of Neurology, University of Washington, Seattle

## Abstract

**Importance:**

Academic detailing (interactive educational outreach) is a widely used strategy to encourage evidence-based prescribing by clinicians.

**Objective:**

To evaluate academic detailing programs targeted at improving prescribing behavior and describe program aspects associated with positive outcomes.

**Evidence Review:**

A systematic search of MEDLINE from April 1, 2007, through December 31, 2022, was performed for randomized trials and nonrandomized studies of academic detailing interventions to improve prescribing. Academic detailing was defined as evidence-based medication education outreach delivered interactively to individuals or small groups of prescribers. Only studies that measured prescribing outcomes were included. Two investigators independently assessed studies for risk of bias using validated assessment tools. Among all studies rated as having low risk of bias and randomized trials rated as having moderate risk of bias, the absolute change in the proportion of patients using the targeted medications and the proportion of studies that led to significant changes in 1 or more prescribing outcome were determined. The data analysis was performed between January 25, 2022, and November 4, 2024.

**Findings:**

The 118 studies identified varied by setting (eg, inpatient, outpatient) and academic detailing delivery (eg, individual vs groups of prescribers). The most common therapeutic targets were antibiotic overuse (32 studies [27%]), opioid prescribing (24 studies [20%]), and management of mental health conditions (16 studies [14%]) and cardiovascular disease (13 studies [11%]). Most studies (66 [56%]) combined academic detailing with other interventions (eg, audit and feedback, electronic health record reminders). Among 36 studies deemed to have the lowest risk of bias, 18 interventions (50%; 95% CI, 33%-67%) led to significant improvements in all prescribing outcomes, and 7 (19%; 95% CI, 8%-36%) led to significant improvements in 1 or more prescribing outcomes. The median absolute change in the proportion of patients using the targeted medication or medications was 4.0% (IQR, 0.3%-11.3%) in the intended direction.

**Conclusions and Relevance:**

In this systematic review of academic detailing interventions addressing evidence-based prescribing, most interventions led to substantial changes in prescribing behavior, although the quality of evidence varied. These findings support the use of academic detailing to bring about more evidence-based prescribing in a variety of clinical settings.

## Key Points

**Question:**

Is academic detailing (interactive educational outreach to clinicians) associated with evidence-based prescribing?

**Findings:**

In this systematic review of 118 randomized trials and nonrandomized studies, clinical setting and delivery of academic detailing interventions varied widely. Among 36 studies deemed to have the lowest risk of bias, 25 (69%) showed significant improvements in at least 1 prescribing behavior, and the median proportion of patients who used the targeted medications changing by 4.0% in the intended direction.

**Meaning:**

These findings suggest that academic detailing can increase evidence-based prescribing in a variety of clinical settings.

## Teaser

This systematic review examines academic detailing programs targeted at improving prescribing behavior and describes program aspects associated with positive outcomes.

## Introduction

Since the 1980s, academic detailing has been an effective strategy to encourage evidence-based prescribing by clinicians.^1,2^ The term academic detailing derives from the delivery of clinical content based only on unbiased noncommercial sources and expert reviews of the medical literature, generally from academic sources. The approach was adapted from the highly effective in-person, 1-on-1 interactive sessions offered by pharmaceutical sales representatives (detailers).^3^ Academic detailing involves interactive educational outreach to clinicians to help them understand and incorporate high-quality evidence into their prescribing decisions.

The purpose of academic detailing is to overcome several obstacles to evidence-based prescribing, including the difficulty of staying abreast of many new drugs and enormous amounts of new evidence while maintaining an active medical practice. For example, 188 new clinical practice guidelines were published in 2022 alone.^4^ Academic detailing is also used to efficiently provide practitioners with current overviews of the best ways to diagnose and manage common clinical problems. A major rationale for its implementation is that practice-improving information is most likely to change behavior if the presentation is based on a given clinician’s particular knowledge, beliefs, and practice. Ideally, through an interactive discussion with an individual clinician, the educator can gain necessary knowledge of the practitioner’s needs, understanding, and attitudes and thus be able to provide a tailored presentation. While the pharmaceutical industry has long used such detailing approaches, their financial interests are inextricably aligned with increasing the use of newer, high-cost medications. In contrast, academic detailing programs run by nonprofit and academic teams are focused on improving prescribing practices based on the best available evidence and thus can act as a counterweight to commercial sales strategies to help clinicians stay abreast of the ever-expanding amount of evidence.

Many previous studies have found that educational interventions that use academic detailing may improve prescribing practices in a variety of settings. In 2007, the Cochrane Collaboration corroborated the association of academic detailing with optimizing medication use by conducting a meta-analysis of 69 randomized trials involving more than 15 000 health professionals.^5^ The interventions that targeted prescribing behaviors had the most consistent effect, leading to a median absolute change in prescribing outcomes of 5.6% in the intended direction. Since the original randomized trials documenting this approach,^1,2^ academic detailing programs have expanded in the US and globally, including a sizeable nationwide program run by the US Department of Veterans Affairs.^6^

Given the substantial investment in academic detailing programs and their considerable potential to improve prescribing behavior, it is imperative to understand when and how to use these interventions most effectively. Despite evolution in how academic detailing is operationalized in the era of electronic health records and access to real-time prescribing data, the last systematic review of academic detailing was published 17 years ago.^5^ To address this gap in the literature, we performed a systematic review of studies investigating the association of academic detailing interventions with targeted prescribing behavior published between 2007 and 2022.

## Methods

For this systematic review, we performed a MEDLINE search to identify research studies of academic detailing interventions related to prescribing from April 1, 2007, through December 31, 2022. We chose a start date of April 2007 to follow up on the prior Cochrane review that included studies published through March 2007.^5^ Our analysis follows the Preferred Reporting Items for Systematic Reviews and Meta-Analyses (PRISMA) guidelines for systematic reviews. The review was not preregistered because the search was initially performed as part of a quality improvement project for the Veterans Health Administration.

We identified studies with at least 1 term related to academic detailing or 1 of its synonyms and 1 term related to prescription drugs (eTable 1 in Supplement 1). To account for heterogeneity in how studies define and report these findings, we used a variety of additional keywords and Medical Subject Heading terms. We separately reviewed the references from related meta-analyses and systematic reviews identified in the MEDLINE search to identify additional studies not captured by our search terms.

For each study identified, we performed an initial screening based on the title and excluded studies that did not describe an intervention (eg, opinion articles, survey studies) or those that described interventions not aimed at prescribers (eg, patient education) or were not focused on prescribing (eg, use of screening tests, lifestyle modification). Among studies that met the initial screening criteria, we reviewed each abstract and, if needed, the entire article to determine whether a study described an educational intervention targeted toward prescribers (ie, not patients or students) that used an academic detailing approach designed to improve prescribing. We defined academic detailing as evidence-based education delivered interactively (ie, not lectures or prerecorded videos) to individuals or small groups of prescribers. We included studies only if they measured prescribing outcomes, excluding those that reported only prescriber perceptions.

### Study Characteristics

For each study that met the inclusion criteria, we extracted study design (randomized vs nonrandomized), setting (eg, inpatient, outpatient), geographic location, therapeutic area (eg, opioids, antibiotics, chronic disease management), qualifications of the practitioners who delivered the intervention (clinician vs trained nonclinician), and the method of academic detailing delivery (ie, meetings with individual prescribers, groups of prescribers, or both). We also identified whether academic detailing was accompanied by any additional interventions, including audit and feedback (ie, providing prescribers with data on their own prescribing), decision support tools (eg, electronic health record messages triggered by prescribing certain medications), and patient education (eg, letters, brochures).

### Risk-of-Bias Assessment

For each study that met the inclusion criteria, we performed a risk-of-bias assessment. For randomized trials, we used version 2 of the Cochrane risk-of-bias tool (RoB 2), and for nonrandomized trials, we used the Risk of Bias in Nonrandomized Studies of Interventions (ROBINS-I) tool.^7,8^ These validated risk-of-bias tools are among the most commonly used in academic systematic reviews and meta-analyses.^9,10,11^ Each study was assessed along several domains of potential bias, including participant selection and recruitment, missing data, and outcome selection and measurement. For randomized trials, each domain was rated as low risk of bias, some concern about bias, or high risk of bias. For nonrandomized studies, domains were rated as having low, moderate, serious, or critical risk of bias.

The risk-of-bias assessments for each domain were combined to obtain an overall risk of bias for each study. In many cases, the domain with the highest risk of bias determined the overall risk of bias, but in some cases, studies with some concerns or moderate risk in a few domains were determined to have an overall low risk of bias based on a qualitative assessment of the source of the potential bias. Each study was assessed independently by 2 of 3 authors (A.C., D.T., or S.R.). Disagreements on domain-specific and overall risk of bias were adjudicated by the third reviewer and resolved through iterative discussion.

### Prescribing Outcomes

Because of the substantial heterogeneity in study methods and outcomes reporting, we did not perform a formal meta-analysis, but we did summarize results from the subset of studies that had the lowest risk of bias. This summary included randomized trials rated as low risk or as having some concerns (ie, moderate risk) and nonrandomized studies rated as low risk of bias. In studies with a higher risk of bias, outcomes may be exaggerated.^12^ We summarized each study’s objective, its primary outcome measurement, and its key results.

From each study, we extracted changes in outcomes that measured the proportion or annual rate of patients prescribed specific medications targeted by the intervention. We focused only on primary outcomes unless the primary outcomes were unrelated to prescribing behavior, in which case we included secondary prescribing outcomes. For each outcome, we determined whether there was a significant change following the intervention based on the study authors’ statistical analysis. We also calculated the absolute changes (ie, risk difference) for the intervention group compared with the control group. Because some academic detailing interventions sought to increase prescribing of certain medications and some were intended to decrease prescribing, we standardized values such that positive differences were in the intended direction.

### Statistical Analysis

The data were analyzed between January 25, 2022, and November 4, 2024. We compared differences in the proportion of prescribing outcomes with significant changes by study characteristics using Fisher exact tests. Similar to a previous Cochrane systematic review, we summarized the effect of the interventions by calculating the median and interquartile range for absolute changes in prescribing.^5^ We compared differences in the absolute changes by study characteristics using Wilcoxon rank sum and Kruskal-Wallis tests. Statistical tests were deemed significant at a 2-sided *P* < .05, and analyses were performed using SAS, version 9.4 (SAS Institute Inc).

## Results

**Figure 1.**
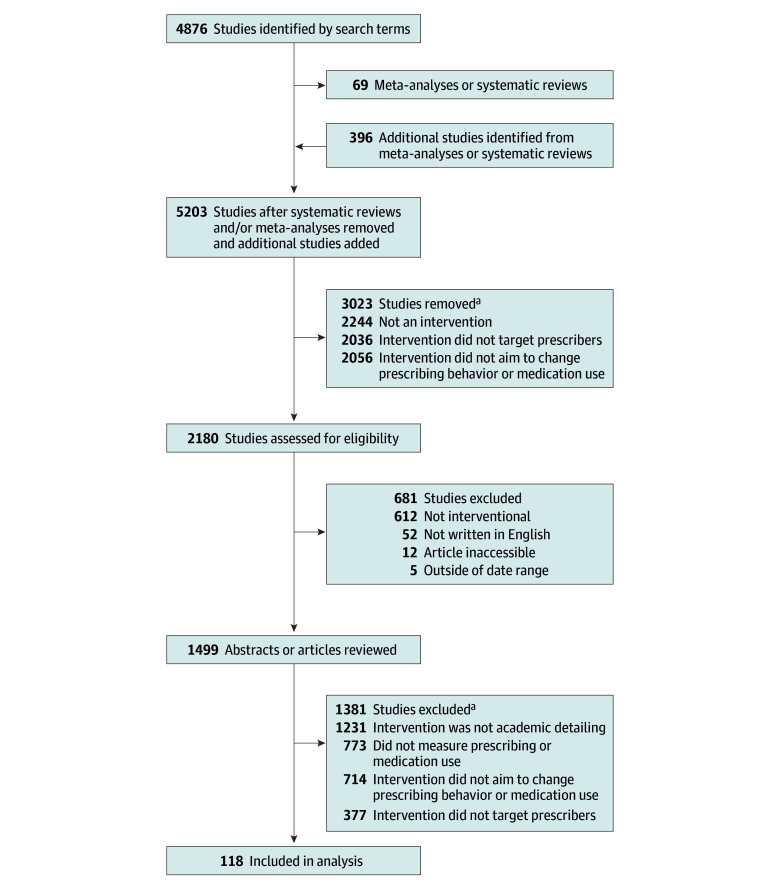
Study Flow Diagram ^a^Could meet multiple exclusion criteria.

We initially identified 5203 studies, including 4876 from our MEDLINE search terms and an additional 396 studies from meta-analyses or systematic reviews (Figure 1). Of these initial studies, 1499 (29%) described an intervention. Ultimately, 118 (8%) met all inclusion criteria and were included in the systematic review.^13,14,15,16,17,18,19,20,21,22,23,24,25,26,27,28,29,30,31,32,33,34,35,36,37,38,39,40,41,42,43,44,45,46,47,48,49,50,51,52,53,54,55,56,57,58,59,60,61,62,63,64,65,66,67,68,69,70,71,72,73,74,75,76,77,78,79,80,81,82,83,84,85,86,87,88,89,90,91,92,93,94,95,96,97,98,99,100,101,102,103,104,105,106,107,108,109,110,111,112,113,114,115,116,117,118,119,120,121,122,123,124,125,126,127,128,129,130^ The most common reasons for exclusion were that an intervention was not considered to be academic detailing (1231 [89%]), did not measure prescribing behaviors or medication use (773 [56%]), and/or did not target prescribing behaviors (714 [52%]); some studies were excluded for multiple reasons.

Study characteristics are summarized in Table 1, and details about each study are shown in eTable 2 in Supplement 1. Overall, 38 (32%) were randomized trials, 24 (20%) were nonrandomized studies and included data from an external control group (historical or contemporary), and 56 (47%) were nonrandomized studies and did not include a control group (eg, baseline to follow-up studies). Most studies (82 [69%]) were conducted in outpatient settings, and 64 (54%) were performed in North America. Most studies (94 [80%]) used academic detailers who were clinicians (eg, physicians, pharmacists, nurses), although 17 (14%) did not specify who delivered the academic detailing intervention. The studies included a mix of interventions with individual clinicians (53 [45%]) and groups of prescribers (33 [28%]); 18 (15%) included both individual and group meetings, and 14 (12%) did not specify how the academic detailing was delivered. The most common therapeutic areas were overprescribing of antibiotics (32 [27%]), opioid prescribing (24 [20%]), and prescribing for mental health conditions (16 [14%]) and cardiovascular disease (13 [11%]). More than one-half of the studies (66 [56%]) combined academic detailing with other types of interventions.

**Table 1.** Characteristics of Included Studies

Characteristic	No. of studies (%)		
	Lowest risk of bias (n = 36)	All others (n = 82)	All studies (N = 118)
Design			
Randomized trial	32 (89)	6 (7)	38 (32)
Nonrandomized study with external control	3 (8)	21 (26)	24 (20)
Nonrandomized study without external control	1 (3)	55 (67)	56 (47)
Setting			
Outpatient	26 (72)	56 (68)	82 (69)
Inpatient hospital	4 (11)	16 (20)	20 (17)
Nursing home	5 (14)	5 (6)	10 (8)
Emergency department	1 (3)	3 (4)	4 (3)
Multiple sites	0	2 (2)	2 (2)
Geographic region			
North America	14 (39)	50 (61)	64 (54)
Europe	17 (47)	14 (17)	31 (26)
Australia	3 (8)	13 (16)	16 (14)
Asia	1 (3)	5 (6)	6 (5)
South America	1 (3)	0	1 (1)
Academic detailer qualifications			
Clinicians (eg, physicians, pharmacists, nurses)	31 (86)	63 (77)	94 (80)
Trained nonclinicians	2 (6)	5 (6)	7 (6)
Not specified^a^	3 (8)	14 (17)	17 (14)
Method of academic detailing delivery			
Meetings with individual prescribers	14 (39)	39 (48)	53 (45)
Meetings with groups of prescribers	12 (33)	21 (26)	33 (28)
Individual and group meetings	7 (19)	11 (13)	18 (15)
Not specified^a^	3 (8)	11 (13)	14 (12)
Therapeutic target			
Antibiotics	9 (25)	23 (28)	32 (27)
Opioids	2 (6)	22 (27)	24 (20)
Mental health (eg, depression, antipsychotics)	6 (17)	10 (12)	16 (14)
Cardiovascular (eg, lipids, heart failure, atrial fibrillation, hypertension)	6 (17)	7 (9)	13 (11)
Polypharmacy or deprescribing	3 (8)	8 (10)	11 (9)
Other chronic diseases (eg, diabetes, kidney disease, osteoporosis)	2 (6)	5 (6)	7 (6)
Vaccines	2 (6)	1 (1)	3 (3)
Drug interactions or renal dosing	0	2 (2)	2 (2)
Other^b^	3 (8)	3 (4)	6 (5)
Multiple areas	3 (8)	1 (1)	4 (3)
Cointerventions			
Audit and feedback	16 (44)	22 (27)	38 (32)
Interactive educational modules	7 (19)	5 (6)	12 (10)
EHR decision support	7 (19)	10 (12)	17 (14)
Patient education	6 (17)	14 (17)	20 (17)
Practice facilitation	5 (14)	11 (13)	16 (14)
Mailings or other follow-up	5 (14)	6 (7)	11 (9)
≥1 Of the above cointerventions	26 (72)	40 (49)	66 (56)

Abbreviation: EHR, electronic health record.

^a^ Information could not be determined from the study article after review by multiple authors (E.D., D.T., P.F.).

^b^ Other included the use of nonsteroidal anti-inflammatory drugs, management of pediatric diarrhea, and use of oxytocin during obstetric deliveries.

### Risk of Bias

**Figure 2.**
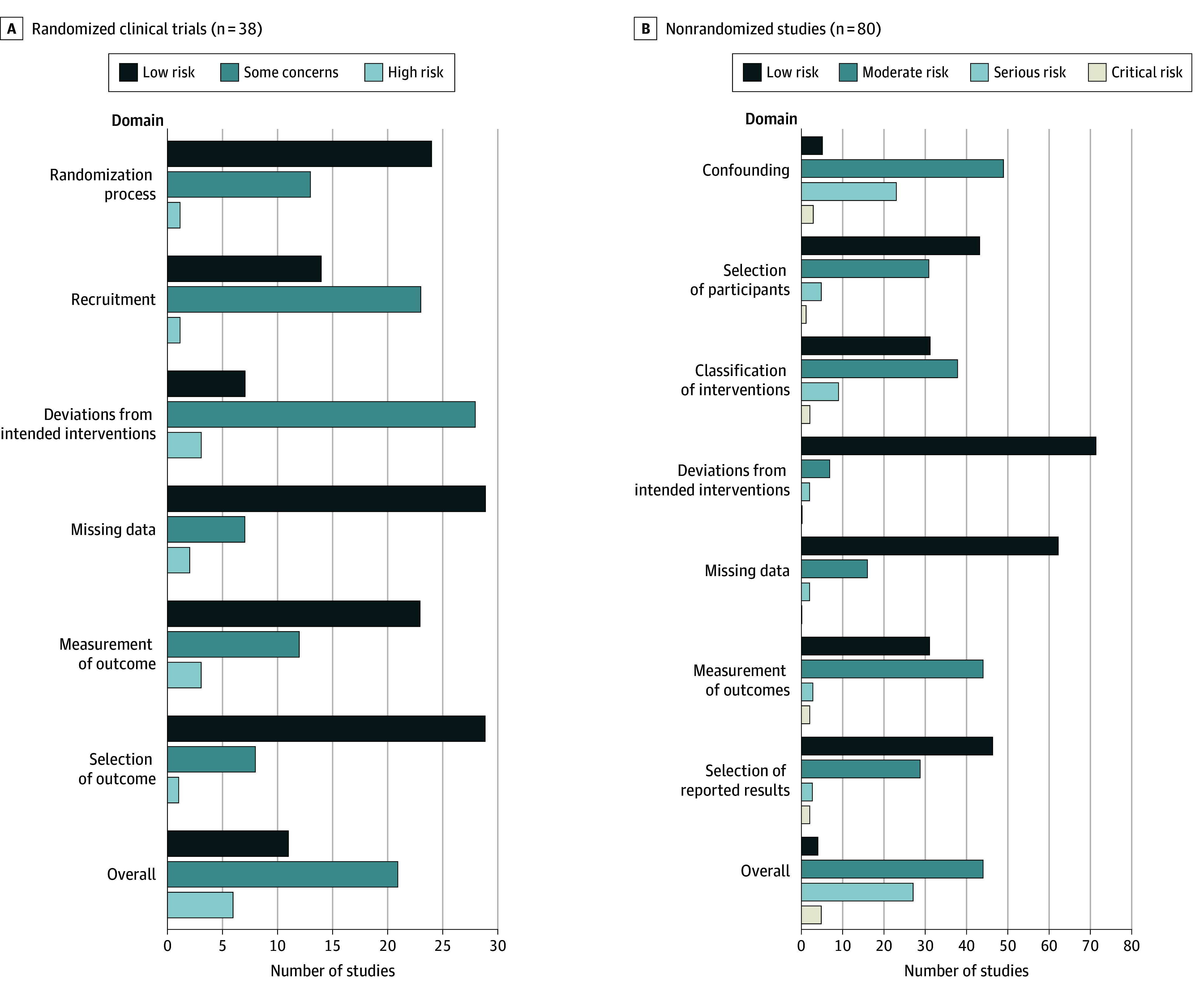
Risk-of-Bias Assessment Ratings

Overall, 11 of the 38 (29%) randomized trials^13,14,15,16,17,18,19,20,21,22,23^ were rated as having a low risk of bias, 21 (55%) had some concern,^28,29,30,31,32,33,34,35,36,37,38,39,40,41,42,43,44,45,46,47,48^ and 6 (16%) had a high risk of bias^52,55,56,63,65,109^ (Figure 2A). The greatest sources of bias were deviation from intended interventions (7 [18%] low risk)^15,16,17,18,19,20,35^ and recruitment (14 [37%] low risk).^13,16,18,19,20,21,22,23,28,31,33,36,38,65^ Of the 80 nonrandomized studies, 4 (5%) were rated as having a low risk of bias,^24,25,26,27^ 44 (55%) as a moderate risk of bias,^49,50,54,57,58,59,64,67,68,69,70,71,73,75,76,77,78,81,83,84,85,86,90,91,92,95,96,97,99,101,102,106,107,108,114,115,116,117,120,121,124,125,127,129^ 27 (34%) as a high risk of bias,^53,60,61,62,66,74,79,80,87,88,89,93,94,98,100,103,105,108,111,112,118,119,122,123,124,128,130^ and 5 (6%) as a critical risk of bias^51,72,82,104,113^ (Figure 2B). Only 5 (6%) were deemed to have a low risk of bias due to confounding.^24,59,64,112,120^ In 6 other domains, the number with a low risk of bias ranged from 31 (39%) to 71 (89%).

### Lower Risk-of-Bias Studies

Characteristics and key findings for the 11 randomized trials^13,14,15,16,17,18,19,20,21,22,23^ and 4 nonrandomized studies^24,25,26,27^ that were rated as having low risk of bias and the 21 randomized trials^28,29,30,31,32,33,34,35,36,37,38,39,40,41,42,43,44,45,46,47,48^ with moderate risk of bias are shown in Table 2. Two of the nonrandomized studies assessed the same intervention at different intervals of follow-up; these were treated separately for the analysis but are shown together in Table 2.^26,27^ Twenty-six of the low-risk-of-bias studies (72%) were conducted in the outpatient setting.^14,15,16,17,18,19,20,21,22,23,24,25,29,30,31,32,33,34,36,37,39,40,41,43,44,45^ Seventeen (47%) were performed in Europe,^15,16,17,20,21,23,24,29,30,31,32,40,41,43,44,45,48^ and 14 (39%) in North America.^14,19,22,25,28,33,34,36,37,38,39,42,46,47^ Most studies (31 [86%]) used academic detailers who were clinicians (ie, physicians, pharmacists, nurses).^13,14,16,17,18,19,20,21,22,23,24,25,26,27,28,29,30,31,33,34,35,38,39,41,42,43,44,45,46,47,48^ Academic detailers met with individual clinicians in 14 studies (39%)^13,18,19,20,22,24,25,26,27,28,31,36,40,45^ and groups of clinicians in 12 studies (33%).^14,15,16,17,21,29,32,33,34,35,43,48^ The most common therapeutic areas targeted by the lower-risk-of-bias studies were antibiotic prescribing (9 [25%])^15,17,25,28,38,39,40,42,48^ and management of cardiovascular (6 [17%])^14,16,20,36,41,43^ or mental health (6 [17%])^26,27,31,33,46,47^ conditions.

**Table 2.** Characteristics and Primary Results for Academic Detailing Studies Deemed as Having the Lowest Risk of Bias

Source	Setting	Study design	Intervention goal	Academic detailing intervention	Cointerventions	Comparator	Primary results^a^
**Randomized clinical trials with low risk of bias**								
Althabe et al,^13^ 2008	19 Hospitals in Argentina and Uruguay	Cluster RCT (by hospital)	Increase use of prophylactic oxytocin during third stage of labor	Individual training of birth attendants by peers	Audit and feedback; posted reminders at clinical sites	Usual care	Prophylactic oxytocin use increased by 67.5% (95% CI, 38.9%-87.1%)
Bertoni et al,^14^ 2009	61 Family and internal medicine practices in North Carolina	Nested RCT (by practice)	Increase adherence to ATP III cholesterol practice guidelines	Introductory lecture followed by 4 1-h group sessions every 6 mo, led by physician and study staff member	Mobile device with cholesterol management tool; review of practice-specific data	Received academic detailing about hypertension guidelines	Appropriate lipid management increased by 9.7% (95% CI, 2.8%-16.6%)
Butler et al,^15^ 2012	263 Primary physicians at 68 practices in the UK	Cluster RCT (by practice)	Decrease antibiotic prescribing	Single practice–based seminar (group) led by a facilitator	Online educational modules; review of practice-specific antibiotic resistance data	Usual care	Total antibiotic dispensing decreased by 4.2% (95% CI, 0.6% -7.7%)
Dreischulte et al,^16^ 2016	34 Primary care practices in Scotland	Stepped wedge cluster RCT (by practice)	Decrease high-risk prescribing of NSAIDs and antiplatelet drugs	1-h Structured educational practice-based session by a pharmacist	Prescriber newsletter sent every 8 wk for 48 wk; EHR informatics tool; small financial incentive for chart review	Preintervention period	Targeted high-risk prescribing decreased by 1.5% (AOR, 0.63; 95% CI, 0.57-0.68)
Gjelstad et al,^17^ 2013	79 General practitioner groups in Norway	Cluster RCT (by practice)	Decrease antibiotic prescribing for acute respiratory tract infections	2 Group visits with a trained physician-academic detailer	Audit and feedback	Similar intervention targeted at decreasing inappropriate medications for older patients	Proportion of acute respiratory tract infection episodes with prescribed antibiotics decreased by 2.8% (AOR, 0.72; 95% CI, 0.61-0.84)
Khanal et al,^18^ 2013	235 Primary care providers in Nepal	Cluster RCT (by prescriber)	Increase adherence to pediatric diarrhea treatment guidelines	Four <30-min monthly individual sessions by trained academic detailers	None	Usual care	Adherence to guidelines increased by 48.5% (*P* < .001)
Liebschutz et al,^19^ 2017	53 Primary care physicians in Boston, Massachusetts	Cluster RCT (by prescriber)	Increase adherence to opioid prescribing guidelines and reduce early refills	Single individual session with physician-opioid prescribing expert	Nurse care manager for opioid prescription monitoring; electronic registry and reports integrated with EHR	Electronic registry alone	Guideline-concordant prescribing increased by 29.1% (AOR, 6.0; 95% CI, 3.6-10.2); no change in early refills
Lowrie et al,^20^ 2014	31 Primary care practices in Scotland	Cluster RCT (by practice)	Increase statin prescribing among patients with vascular disease	Individual sessions with pharmacist every 4 mo for 1 y	Pharmacist case manager who identified patients and created custom patient materials	Usual care	Proportion achieving cholesterol target increased by 8.3% (AOR, 1.11; 95% CI, 1.00-1.23)
Magrini et al,^21^ 2014	1737 Primary care practitioners at 115 practices in Italy	Cluster RCT (by practice)	Trial 1, increase appropriate prescribing for benign prostate hyperplasia or osteoporosis; trial 2, decrease prescribing of barnidipine or prulifloxacin	Biannual 3-4-h practice-level small-group educational meetings led by a trained pharmacist and a physician	None	Each intervention served as the comparator for the other intervention within in the same trial	Trial 1, ratio of brand-name alfuzosin to tamsulosin and terazosin prescribing decreased by 8.5% (95% CI, 0.7%-16.9%), with no change in 3 other primary outcomes; trial 2, prescribing rates decreased by 11.1% (95% CI, 0.5%-22.2%) for prulifloxacin and 9.8% (95% CI, 1.9%-18.2%) for barnidipine^b^
Solomon et al,^22^ 2007	828 Primary care practitioners in Pennsylvania	Cluster RCT (by prescriber)	Increase screening and treatment of osteoporosis	Single academic detailing session led by trained pharmacists and nurses	Educational letters mailed to patients	Usual care	Composite outcome (bone mineral density testing or initiating osteoporosis medication) no different in intervention or control (relative risk, 1.04; 95% CI, 0.85-1.26)
Willis et al,^23^ 2020	178 National Health Service general practices in England	2 Parallel cluster RCTs (by practice)	Trial 1, increase diabetes control or decrease risky prescribing of NSAIDs and antiplatelets; trial 2, increase blood pressure control or increase anticoagulant use in atrial fibrillation	30-min Individual or group pharmacist-led academic detailing session with optional follow-up session	Audit and feedback; automated prompts for high-risk prescribing behaviors	Each intervention served as the comparator for the other intervention within the same trial	Proportion of patients with risky NSAID and antiplatelet prescribing decreased by 1.1% (AOR, 0.82; 95% CI, 0.67-0.99); no change in proportion achieving diabetes control, blood pressure control, or anticoagulation in atrial fibrillation
**Nonrandomized studies with low risk of bias**								
Langaas et al,^24^ 2019	212 General practitioners in 2 cities in Norway	Interrupted time series	Decrease diclofenac use and increase naproxen use	20-min Individual academic detailing visit by a trained pharmacist or consultant	None	(1) Two similarly sized cities in Norway; (2) entire country of Norway	Monthly rate of diclofenac prescribing decreased by 1.40 (95% CI, 0.58-2.22) per 1000 inhabitants in 1 city and 1.12 (95% CI, 0.35-1.88) per 1000 inhabitants in the other city compared with all of Norway
Portman et al,^25^ 2020	Veterans Affairs Healthcare System in Pennsylvania	Interrupted time series	Decrease fluoroquinolone prescribing	Single individual 15-30-min session with a pharmacist or nurse	(1) Audit and feedback; (2) change in practice documentation requirements to justify fluoroquinolone initiation	None	Fluoroquinolone prescription incidence rate level decreased by 2.0 (95% CI, 1.4-2.6) per 1000 and slope decreased by 0.013 (95% CI, 0.007-0.018) per 1000
Westbury et al,^26^ 2010; Westbury et al,^27^ 2011	25 Nursing homes in Australia	Nonrandomized controlled intervention	Decrease benzodiazepine and antipsychotic use	Single group session with researcher	(1) 2-d Training for pharmacists; (2) 2 nurse training sessions; (3) posted educational signs	Usual care	Decrease in proportion using benzodiazepines (7.5%; *P* < .005) and antipsychotics (4.6%; *P* < .05) after 6 mo; sustained for benzodiazepines (6.0%) but not antipsychotics after 18 mo
**Randomized trials with some concerns for risk of bias**								
Camins et al,^28^ 2009	Internal medicine wards at an academic hospital in Atlanta, Georgia	RCT (by team)	Increase proportion of appropriate antimicrobial use	Structured feedback by antimicrobial use team via telephone call or face-to-face meeting	Pocket-sized guidelines for clinicians	Use of indication-based antimicrobial guidelines	Higher proportion of empirical (78% vs 58%), definitive (82% vs 43%), and end (94% vs 70%) antimicrobial use deemed appropriate
Clyne et al,^29^ 2015; Clyne et al,^30^ 2016	21 Primary care practices in Ireland	Cluster RCT (by practice)	Decrease potentially inappropriate prescribing in older adults	30-min Session with a pharmacist	Web-based treatment algorithms for each patient; patient information leaflets	Usual care; list of patients with potentially inappropriate prescribing	Lower proportion of patients with potentially inappropriate prescribing (52% vs 77%; *P* = .02) and mean number of potentially inappropriate prescriptions (0.70 vs 1.18; *P* = .02) in the intervention group; results sustained after 12 mo
Eccles et al,^31^ 2007	72 Primary care practices in the UK	Cluster RCT (by practice)	Increase use of cost-effective antidepressants over less cost-effective classes	One or two 20-45-min visits by a pharmacist 4-6 wk apart	None	Mailed guideline materials	No difference in the prescribing rates of different antidepressant classes between the intervention and control groups
Enriquez-Puga et al,^32^ 2009	28 Primary care practices in the UK	Cluster RCT (by practice)	Decrease use of broad-spectrum antibiotics; increase use of guideline-recommended antidepressants	Two 20-40-min group sessions 6 mo apart	Flowcharts and algorithms provided to clinicians	Interventions for depression and antibiotics served as control groups for one another	The group receiving the depression intervention had higher use of the recommended antidepressant lofepramine (rate ratio, 2.85; 95% CI, 1.92-4.34); there were no changes in antibiotic use
Fortuna et al,^33^ 2009	14 Internal medicine practices in the US	Cluster RCT (by practice)	Decrease the prescribing of heavily marketed hypnotic medications	One 45-min group educational session led by an experienced internist	Computerized prescribing alerts	Usual care; computerized alerts only	The proportion of prescriptions for heavily marketed hypnotics was lower than usual care (risk ratio, 0.74; 95% CI, 0.58-0.97); results were similar for the alert-only group
Franzini et al,^34^ 2007	189 Pediatric and family medicine practices around Houston, Texas	Cluster RCT (by practice)	Increase guideline-concordant childhood immunization rates	1-h Group educational session with a trained physician, nurse, and office manager	Mailed educational materials to each practice monthly for 6 mo after the presentation	Usual care	No difference in immunization rates between the intervention and control groups
Hopkins et al,^35^ 2020	Surgical units at a single teaching hospital in Melbourne, Australia	Pragmatic cluster RCT (by unit)	Decrease postsurgical discharge prescribing of slow-release opioids	Single 30-min face-to-face group education session with a pharmacist, with presentation slides shared by email	Presentation slides sent to all eligible clinicians	Routine continuing education and institutional resources	The proportion of patients prescribed slow-release opioids decreased by 15% in the intervention group vs 6.4% in the control group (*P* < .001)
Kapoor et al,^36^ 2020	79 Primary care clinicians in Massachusetts	Cluster RCT (by clinician)	Increase anticoagulation use for atrial fibrillation	Initial remote or face-to-face 30-60-min interaction, and a single 15-30-min follow-up session	Monthly reports of clinician-specific data; electronic messages before appointments with eligible untreated patients	Usual care	No significant difference between the change in anticoagulation use for the intervention and control groups
Ly et al,^37^ 2015	6 Pediatric clinics in San Diego, California	Cluster RCT (by clinic)	Increase childhood vaccination rates	Single educational visit with each clinic	Clinic-specific baseline immunization rates; assistance in implementing workflow changes	Usual care	Immunization rate increased from 55.5% to 63.1% in the intervention group (*P* = .03) vs 73.0% to 77.4% in the control group (*P* = .13)
Metlay et al,^38^ 2007	16 VA and non-VA emergency departments in US cities	Cluster RCT (by site)	Decrease antibiotic use for acute respiratory infections	Multiple individual and group sessions by a trained onsite clinical leader	Patient education brochures and video kiosk in the waiting room	Usual care	Adjusted antibiotic use decreased from 59% to 49% in the intervention group vs 45% to 43% in the control group
Mortrude et al,^39^ 2021	5 VA primary care clinics in Wisconsin	Stepped wedge trial	Decrease antibiotic use for acute respiratory infections	Single in-person educational session and distributed informational pocket cards	Report cards given to clinicians 3-7 d before academic detailing session; clinical decision support within EHR; patient education brochures	Baseline and follow-up intervention	Antibiotic prescribing decreased from 56% vs 49% after implementation of the intervention (not significant)
Naughton et al,^40^ 2009	98 Primary care clinics in Ireland	Cluster RCT (by clinic)	Decrease antibiotic prescribing	15-30-min Outreach visit from the research coordinator with individualized prescribing feedback	Mailed report of clinician-level antibiotic prescribing data	Mailed report only	No significant change in antibiotic prescribing in the intervention group compared with the control group
Naughton et al,^41^ 2007	98 Primary care clinics in Ireland	Cluster RCT (by clinic)	Increase use of preventive cardiovascular medications (statins and antiplatelets)	15-30-min Outreach visit from the research coordinator with individualized prescribing feedback	Mailed report of clinician-level prescribing data	Mailed report only	No significant change in cardiovascular drug prescribing in the intervention group compared with the control group
Pasay et al,^42^ 2019	42 Nursing homes in Alberta, Canada	Cluster RCT (by nursing home)	Decrease antimicrobial prescribing for urinary tract infections	Individual and small-group sessions with a pharmacist	Clinical decision-making checklist tool	Usual care	Antimicrobial prescriptions decreased from 3.1 to 2.5 per 1000 resident days in the intervention group vs 3.0 to 3.3 per 1000 resident days in the control group (*P* < .001)
Peters-Klimm et al,^43^ 2008	37 Primary care physicians in Germany	Cluster RCT (by physician)	Increase prescribing of ACE inhibitors or ARBs, β-blockers, and aldosterone antagonists for heart failure	Single didactic session plus 4 repeated interdisciplinary educational sessions and individual feedback sessions	None	Single didactic session only	Significant increase in the use of aldosterone antagonists in the intervention group (AOR, 3.5; 95% CI, 1.1-11.1); for ACE inhibitors or ARBs and β-blockers, there was no difference in use, but the intervention group had more patients at target dose
Pinto et al,^44^ 2018	38 Primary care clinics in Lisbon, Portugal	Cluster RCT (by clinic)	Decrease COX-2 inhibitor prescriptions; increase omeprazole prescriptions	15-20-min Individual educational session	None	Usual care	No significant difference between intervention and control groups in omeprazole and COX-2 inhibitor prescription rates
Rognstad et al,^45^ 2013	465 General practitioners in Norway	Cluster RCT (by continuing medical education group)	Decrease potentially inappropriate prescriptions for older patients	2 Individual sessions 2 mo apart, followed 3 mo later by a group full-day workshop	Audit and feedback	Opposite arm targeting respiratory tract antibiotic prescribing	The proportion of patients exposed to a potentially inappropriate prescription decreased by 1.6% in the intervention group compared with the control group
Tadrous et al,^46^ 2020	40 Nursing homes in Ontario, Canada	Cluster RCT (by nursing home)	Decrease prescribing of continuous antipsychotics	Individual meeting between nurses or pharmacists and nursing home practitioners	None	Usual care	No significant change in the proportion of nursing home residents using daily antipsychotics
Tjia et al,^47^ 2015	42 Nursing homes in Connecticut	Cluster RCT (by nursing home)	Decrease the use of antipsychotics	Individual meeting between nurses or pharmacists and nursing home practitioners	Mailed educational toolkit; quarterly facility-level audit and feedback reports; behavioral management training	Toolkit only; toolkit plus audit and feedback only	No significant difference in antipsychotic use between the study arms
Wathne et al,^48^ 2018	Infectious disease, pulmonary medicine, and gastroenterology wards at 3 hospitals in Norway	Cluster RCT (by ward)	Increase adherence to appropriate empirical antibiotic therapy; decrease days of broad-spectrum antibiotic exposure	Group sessions with infectious disease physicians and pharmacists	None	Usual care; audit and feedback	No significant improvement in adherence to antibiotic guidelines after the intervention compared with control wards

Abbreviations: ACE, angiotensin-converting enzyme; AOR, adjusted odds ratio; ARB, angiotensin receptor blocker; ATP III, Third National Cholesterol Education Program Adult Treatment Panel; COX-2, cyclooxygenase 2; EHR, electronic health record; NSAID, nonsteroidal anti-inflammatory drug; RCT, randomized clinical trial; VA, Veterans Administration.

^a^ Unless otherwise specified, results are reported as the absolute change in prescribing compared with the control group.

^b^ Relative changes because absolute changes were not reported.

In 26 studies (72%),^13,14,15,16,17,19,20,22,23,25,26,27,28,29,30,33,34,35,36,37,38,39,40,41,42,47^ academic detailing was combined with other types of interventions. The most common cointerventions were audit and feedback (16 studies [44%]),^13,14,15,16,17,19,20,23,26,27,36,37,39,40,41^ in which the educator shared recent prescriber- or practice-level prescribing data. Others included noninteractive educational modules given to prescribers (7 [19%])^14,16,19,29,30,42,47^ and automated warnings or reminders in the electronic health record triggered by certain prescribing (7 [19%]).^16,23,25,33,36,37,39^ Six studies (17%) combined academic detailing with educational outreach to patients,^22,29,30,37,38,39^ and 5 (14%) included a nurse or pharmacist care manager embedded in the clinical practices to actively identify patients in need of prescribing change.^16,19,20,37,47^ One study provided a small payment to prescribers for reviewing the records of patients with potentially high-risk prescribing.^16^

### Association of Academic Detailing Interventions With Prescribing Practices

Among the 36 studies rated as having a low risk of bias,^13,14,15,16,17,18,19,20,21,22,23,24,25,26,27,28,29,30,31,32,33,34,35,36,37,38,39,40,41,42,43,44,45,46,47,48^ we identified 67 prescribing outcomes (range, 1-4 per study). Among these outcomes, 36 (54%; 95% CI, 41%-66%) showed a statistically significant change in the desired direction following the intervention (Table 3). A greater likelihood of success was seen with interventions in which detailers met with individual clinicians compared with other interventions (20 of 29 [69%] vs 16 of 38 [42%] outcomes, respectively; *P* = .047), and those that combined academic detailing with at least 1 additional intervention vs academic detailing alone (26 of 38 [68%] vs 10 of 29 [34%] outcomes, respectively; *P* = .007).

**Table 3.** Prescribing Outcomes From 36 Lower-Risk-of-Bias Studies

Study characteristic	No. of significant outcomes of No. of outcomes (%)	*P* value^a^	No. with absolute change^b^	Absolute % change, median (IQR)	*P* value^c^
All studies	36 of 67 (46)	NA	57	4.0 (0.3 to 11.3)	NA
Design					
Randomized trial	30 of 60 (50)	.11	50	4.7 (0.9 to 17.4)	.15
Nonrandomized study	6 of 7 (86)		7	0.2 (0.1 to 6.0)	
Setting					
Outpatient	26 of 50 (52)	.78	40	3.2 (0.4 to 12.0)	.68
Inpatient, nursing home, or emergency department	10 of 17 (48)		17	6.0 (0.1 to 10.0)	
Geographic region					
North America	12 of 24 (50)	.80	23	4.5 (0.1 to 9.9)	.90
Other region	24 of 43 (56)		34	3.4 (0.5 to 17.8)	
Academic detailer qualification					
Clinician	33 of 59 (56)	.46	54	4.7 (0.3 to 12.6)	.31
Other^d^	3 of 8 (38)		3	2.6 (−0.2 to 3.4)	
Detailer meeting type					
Individual clinician	20 of 29 (69)	.047	28	6.8 (0.2 to 20.0)	.20
Other^e^	16 of 38 (42)		29	3.0 (0.5 to 7.2)	
Therapeutic target					
Antibiotics	8 of 11 (73)	.16	10	6.5 (2.6 to 10.0)	.12
Mental health	4 of 14 (29)		13	0.9 (−0.6 to 3.7)	
Cardiovascular	7 of 12 (58)		12	6.5 (1.2 to 15.0)	
Other^f^	17 of 30 (57)		7	4.2 (0.5 to 21.5)	
Cointerventions					
Yes^g^	26 of 38 (68)	.007	36	5.4 (1.3 to 10.6)	.15
None	10 of 29 (34)		21	1.6 (0.0 to 12.6)	

Abbreviation: NA, not applicable.

^a^ *P* values from Fisher exact tests comparing the proportion of significant outcomes by study characteristics.

^b^ Number of studies for which sufficient data were reported to calculate the absolute change in the proportion of patients affected by the targeted prescribing behavior after the intervention compared with the control group.

^c^ *P* values from Wilcoxon rank sum tests or Kruskal-Wallis tests comparing changes in outcomes by study characteristics.

^d^ Includes studies with trained nonclinician detailers and studies where the detailer credentials were not specified.

^e^ Includes studies with group meetings, with both individual and group meetings, and for which the meeting type was not specified.

^f^ Includes studies targeting opioids, polypharmacy or deprescribing, other chronic diseases, vaccines, and nonsteroidal anti-inflammatory drugs; management of pediatric diarrhea; use of oxytocin during obstetric deliveries; and multiple therapeutic areas.

^g^ Includes audit and feedback, interactive educational modules, electronic health record decision support tools, patient education, practice facilitation, or mailing or other clinician follow-up.

Of the 36 studies,^13,14,15,16,17,18,19,20,21,22,23,24,25,26,27,28,29,30,31,32,33,34,35,36,37,38,39,40,41,42,43,44,45,46,47,48^ 18 interventions (50%; 95% CI, 33%-67%) produced significant changes in all measured prescribing outcomes^13,15,16,17,18,20,24,25,26,28,29,30,33,35,37,38,42,45^ and 11 (31%; 95% CI, 16%-48%) did not.^22,31,34,36,39,40,41,44,46,47,48^ Seven studies (19%; 95% CI, 8%-36%) with more than 1 outcome had significant changes in some of the measures.^14,19,21,23,27,32,43^ For example, Willis et al^23^ randomized practices to receive 1 of 4 distinct academic detailing interventions. Only the intervention aimed at reducing potentially risky prescribing of nonsteroidal anti-inflammatory drugs and antiplatelet drugs led to a significant change in prescribing behavior, while interventions aimed at improving control of diabetes or blood pressure and to increase anticoagulant use in atrial fibrillation did not result in significantly improved prescribing outcomes.

We were able to calculate absolute changes in the proportion of patients experiencing the intended prescribing behavior for 57 prescribing outcomes (85%). The median change was 4.0% (IQR, 0.3%-11.3%). There were no significant differences in the postintervention changes in prescribing behavior among studies with varying characteristics (Table 3).

## Discussion

In this systematic review of 118 studies of academic detailing to improve prescribing behavior from 2007 to 2022,^13,14,15,16,17,18,19,20,21,22,23,24,25,26,27,28,29,30,31,32,33,34,35,36,37,38,39,40,41,42,43,44,45,46,47,48,49,50,51,52,53,54,55,56,57,58,59,60,61,62,63,64,65,66,67,68,69,70,71,72,73,74,75,76,77,78,79,80,81,82,83,84,85,86,87,88,89,90,91,92,93,94,95,96,97,98,99,100,101,102,103,104,105,106,107,108,109,110,111,112,113,114,115,116,117,118,119,120,121,122,123,124,125,126,127,128,129,130^ we found substantial variation in settings, therapeutic targets, mode of delivery and whether it was combined with additional interventions (eg, audit and feedback). Among the 36 studies rated as having the lowest risk of bias,^13,14,15,16,17,18,19,20,21,22,23,24,25,26,27,28,29,30,31,32,33,34,35,36,37,38,39,40,41,42,43,44,45,46,47,48^ 69% reported that academic detailing interventions produced significant changes in at least 1 prescribing outcome, with a median 4.0% absolute improvement in the proportion of patients prescribed medications targeted by the interventions. These results suggest that academic detailing can improve prescribing behavior across a wide range of therapeutic areas and clinical settings.

Academic detailing has continued to evolve since the approach was first described several decades ago.^1,2,3^ For example, academic detailing is now most frequently combined with other interventions made possible by technological advancements, such as leveraging prescribers’ own data (ie, audit and feedback) and integrating decision support tools within electronic health records. Concurrent use of prescriber-specific data on medication use and outcomes has been termed interventional pharmacoepidemiology and can be a powerful way to assess, target, and evaluate such educational interventions.^131^ Complex interventions that combine education with other tools could potentially improve the outcomes associated with either intervention alone, but they are also more difficult to implement and evaluate.^132^ We found that most of the highest-quality studies in this review used such multimodal interventions, and studies that combined academic detailing with 1 or more intervention were more likely to report significant improvements in the targeted prescribing behavior compared with studies of academic detailing alone.

Another change over the past few decades has been the rapid rise in technological solutions for accessing up-to-date clinical information, which some have argued might limit the impact of in-person educational iniatives.^133^ Despite this changing landscape, our findings are consistent with the conclusions of the last major systematic review of academic detailing interventions published by the Cochrane Collaboration in 2007, which found that academic detailing interventions led to a median 4.8% change in prescribing behavior.^5^ Together, these findings suggest that individualized educational outreach still plays an important role possibly because prescribers who are in greatest need of medication information may not be the ones who access it most frequently. Notably, although the absolute magnitude of changes is modest, even a shift of a few percentage points in medication use can translate to clinically important changes in care and substantial changes in cost if implemented on a large scale.

We found considerable heterogeneity in study design and quality, measures used, and analytic approaches, which is not surprising because many analyses were designed to evaluate the implementation of actual clinical programs. Although randomized trials are ideal for evaluating any intervention in health care, it is often difficult to implement such study designs in operational settings. These challenges may be mitigated by alternative trial designs, such as stepped wedge trials that randomize the order of a staged intervention rollout.^16,38,134^ Even when studies are well designed and well executed, it may be difficult to interpret and generalize the results from multifaceted complex interventions.^132^

The results of this review may inform best practices for using academic detailing to improve prescribing practices, especially as medication use becomes ever more central to health care choices and outcomes. For example, we found that interventions in which academic detailers met with individual clinicians were more likely to significantly affect prescribing practices compared with those in which detailers met with groups of clinicians. A possible explanation for this finding may be that individual meetings allow detailers to tailor the educational approach and messaging to best address individual clinicians’ prescribing behaviors.^3^ Combining academic detailing with other approaches can also be an important strategy to boost the effectiveness of this intervention. Notably, we found no significant differences in the success of academic detailing across different settings (eg, outpatient vs inpatient) and therapeutic targets, suggesting that the approach can be useful in a variety of contexts.

This systematic review also identified additional opportunities to improve the evidence concerning academic detailing. Many of the studies identified had important methodological limitations, particularly confounding in nonrandomized studies. A substantial minority of studies did not adequately present details of the academic detailing intervention, including who delivered the educational content and how this was done. To inform future programmatic efforts and facilitate improvement and use of best practices, future studies must provide sufficient details about the interventions.

Academic detailing programs have been adopted most intensively in integrated health care systems, such as the Veterans Health Administration system in which there is an alignment of goals across prescribers, pharmacists and pharmacies, hospitals, and the funding mechanism.^6,135^ To the extent that the US or other health care jurisdictions move toward more integrated methods of care delivery and reimbursement, academic detailing may be a practical approach for improving the quality and appropriateness of medication use.

### Limitations

This systematic review has several important limitations. Although we identified studies based on an extensive list of search terms and screened more than 5000 studies, we may not have identified all reported studies of academic detailing due to differences in the terminology used to describe these interventions. Because of substantial variation in study design and how outcomes were reported, we were unable to perform a formal meta-analysis. However, by reviewing outcomes of a subset of lower-risk-of-bias studies, the results provide compelling evidence that academic detailing interventions are likely to lead to substantial improvements in prescribing behaviors over a wide range of settings and clinical topics.

## Conclusions

In this systematic review of 118 studies of academic detailing interventions to improve prescribing, there was substantial variation in the delivery of the intervention and the quality of the evidence. In the 36 studies with the lowest risk of bias, 69% were associated with \significantly improved evidence-based prescribing, with a median change of 4.0%. These findings support the use of academic detailing as a strategy to improve evidence-based prescribing in a variety of clinical settings.

## Notes

Supplement 1. **eTable 1.** MEDLINE Search Terms **eTable 2.** Study Characteristics

Supplement 2.
Data Sharing Statement
