# Supplementary material for: Academic Detailing Interventions and Evidence-Based Prescribing: A Systematic Review
Source: JAMA Netw Open. 2025 Jan 8;8(1):e2453684. doi: 10.1001/jamanetworkopen.2024.53684 (PMC12543401; doi:10.1001/jamanetworkopen.2024.53684)
Supplement: Supplement 1. — eTable 1. MEDLINE Search Terms eTable 2. Study Characteristics [file jamanetwopen-e2453684-s001.pdf]

Supplemental Online Content

Rome BN, Dancel E, Chaitoff A, et al. Academic detailing interventions and evidence-based prescribing: a systematic review. *JAMA Netw Open*. 2025;8(1):e2453684. doi:10.1001/jamanetworkopen.2024.53684

eTable 1. MEDLINE Search Terms

eTable 2. Study Characteristics

This supplemental material has been provided by the authors to give readers additional information about their work.

© 2025 Rome BN et al. *JAMA Network Open.*

eTable 1. MEDLINE Search Terms

	"Academic Detailing Intervention"	"Prescription Drugs"
**Medical Subject Heading Terms (MeSH)**	• Education, Medical, Continuing<br>• Education, pharmacy<br>• Education, continuing<br>• Education, professional	• Inappropriate Prescribing<br>• Potentially Inappropriate Medication List<br>• Practice Patterns, Physicians<br>• Drug Therapy
**Other key words**	• Academic detailing<br>• Outreach education<br>• Educational-outreach<br>• Educational outreach<br>• Educational intervention<br>• Educational program<br>• Peer education<br>• Educational session<br>• Professional education	• Prescription drug<br>• Prescription medication<br>• Prescribing<br>• Prescriptions<br>• Medication use<br>• De-prescribing<br>• Deprescribing<br>• Vaccine<br>• Vaccination<br>• Pharmacological

We searched MEDLINE for studies with at least 1 MeSH term or key word from each column.

© 2025 Rome BN et al. *JAMA Network Open.*

eTable 2. Study characteristics

Ref #	Source	Design [a]	Setting	Geographic region	Delivered by clinician	Method	Therapeutic target [c]	Cointerventions [d]
13	Althabe et al, 2008	RCT	Inpatient	S America	Yes	Individual	Other	AF FU
14	Bertoni et al, 2009	RCT	Outpatient	N America	Yes	Group	Cardiovascular	AF IM
15	Butler et al, 2012	RCT	Outpatient	Europe	No	group	Antibiotics	AF
16	Dreischulte et al, 2016	RCT	Outpatient	Europe	Yes	Group	Cardiovascular	AF IM EHR PF FU
17	Gjelstad et al, 2013	RCT	Outpatient	Europe	Yes	Group	Antibiotics	AF
18	Khanal et al, 2013	RCT	Outpatient	Asia	Yes	Individual	Other	None
19	Liebschutz et al, 2017	RCT	Outpatient	N America	Yes	Individual	Opioids	AF IM PF
20	Lowrie et al, 2014	RCT	Outpatient	Europe	Yes	Individual	Cardiovascular	AF PF
21	Magrini et al, 2014	RCT	Outpatient	Europe	Yes	Group	Chronic	None
22	Solomon et al, 2007	RCT	Outpatient	N America	Yes	Individual	Chronic	PE
23	Willis et al, 2020	RCT	Outpatient	Europe	Yes	Both	Multiple	AF EHR
24	Langaas et al, 2019	C	Outpatient	Europe	Yes	Individual	Other	None
25	Portman et al, 2020	NC	Outpatient	N America	Yes	Individual	Antibiotics	EHR
26	Westbury et al, 2010	C	Nursing home	Australia	Yes	Individual	Mental health	AF
27	Westbury et al, 2011	C	Nursing home	Australia	Yes	Individual	Mental health	AF
28	Camins et al, 2009	RCT	Inpatient	N America	Yes	Individual	Antibiotics	FU
29	Clyne et al, 2015	RCT	Outpatient	Europe	Yes	Group	Polypharmacy	IM PE
30	Clyne et al, 2016	RCT	Outpatient	Europe	Yes	Unknown	Polypharmacy	IM PE
31	Eccles et al, 2007	RCT	Outpatient	Europe	Yes	Individual	Mental health	None
32	Enriquez-Puga et al, 2009	RCT	Outpatient	Europe	Unknown	Group	Multiple	None
33	Fortuna et al, 2009	RCT	Outpatient	N America	Yes	Group	Mental health	EHR
34	Franzini et al, 2007	RCT	Outpatient	N America	Yes	Group	Vaccines	FU
35	Hopkins et al, 2020	RCT	Inpatient	Australia	Yes	Group	Opioids	FU
53	Kapoor et al, 2020	RCT	Outpatient	N America	Unknown	Individual	Cardiovascular	AF EHR
36	Ly et al, 2015	RCT	Outpatient	N America	Unknown	Unknown	Vaccines	AF EHR PE PF
37	Metlay et al, 2007	RCT	Emergency	N America	Yes	Both	Antibiotics	PE
38	Mortrude et al, 2021	RCT	Outpatient	N America	yes	Unknown	Antibiotics	AF EHR PE
39	Naughton et al, 2009	RCT	Outpatient	Europe	No	Individual	Antibiotics	AF
40	Naughton et al, 2007	RCT	Outpatient	Europe	Yes	Both	Cardiovascular	AF
41	Pasay et al, 2019	RCT	Nursing home	N America	Yes	Both	Antibiotics	IM
42	Peters-Klimm et al, 2008	RCT	Outpatient	Europe	Yes	Group	Cardiovascular	None
43	Pinto et al, 2018	RCT	Outpatient	Europe	Yes	Both	Multiple	None

© 2025 Rome BN et al. *JAMA Network Open.*

Ref #	Source	Design [a]	Setting	Geographic region	Delivered by clinician	Method	Therapeutic target [c]	Cointerventions [d]
44	Rognstad et al, 2013	RCT	Outpatient	Europe	Yes	Individual	Polypharmacy	None
45	Tadrous et al, 2020	RCT	Nursing home	N America	Yes	Both	Mental health	None
46	Tjia et al, 2015	RCT	Nursing home	N America	Yes	Both	Mental health	AF IM PF
47	Wathne et al, 2018	RCT	Inpatient	Europe	Yes	Group	Antibiotics	None
54	Abd-Elsayed et al, 2018	NC	Multiple	N America	Unknown	Group	Opioids	EHR
55	Akkawi et al, 2020	NC	Inpatient	Asia	Yes	Both	Polypharmacy	IM
56	Akter et al, 2009	C	Inpatient	Asia	Unknown	Group	Antibiotics	None
57	Altiner et al, 2007	RCT	Outpatient	Europe	Yes	Individual	Antibiotics	PE
58	Baandrup et al, 2010	C	Outpatient	Europe	No	Group	Mental health	EHR
59	Baum et al, 2010	NC	Inpatient	Europe	Yes	Group	Interactions	None
60	Behar et al, 2017	RCT	Outpatient	N America	Unknown	Individual	Opioids	None
61	Bhunia et al, 2010	RCT	Inpatient	Asia	Yes	Group	Other	None
62	Bounthavong et al, 2022	NC	Outpatient	N America	Yes	Individual	Opioids	None
63	Bounthavong et al, 2017	NC	Outpatient	N America	Yes	Individual	Opioids	None
64	Bounthavong et al, 2020	C	Outpatient	N America	Yes	Individual	Mental health	None
65	Bounthavong et al, 2019	NC	Outpatient	N America	Yes	Individual	Opioids	None
66	Bounthavong et al, 2020	NC	Outpatient	N America	Yes	Individual	Opioids	None
67	Bounthavong et al, 2021	C	Outpatient	N America	Yes	Individual	Opioids	None
68	Bregnhøj et al, 2009	RCT	Outpatient	Europe	Yes	Both	Polypharmacy	None
69	Brunette et al, 2018	NC	Outpatient	N America	Yes	Group	Polypharmacy	AF
70	Bruyndonckx et al, 2018	RCT	Outpatient	Europe	Unknown	Unknown	Opioids	None
71	Buising et al, 2008	NC	Inpatient	Australia	Yes	Individual	Antibiotics	None
72	Dehn et al, 2018	NC	Outpatient	Asia	Unknown	Both	Antibiotics	AF IM FU
73	Dieujuste et al, 2020	NC	Emergency	N America	Yes	Individual	Opioids	AF EHR PF
74	Donaldson et al, 2017	NC	Emergency	Australia	Yes	Individual	Opioids	None
75	Doubova et al, 2010	C	Outpatient	N America	Yes	Both	Opioids	None
76	Doyon et al, 2009	NC	Inpatient	N America	Yes	Group	Antibiotics	None
77	Duff et al, 2013	NC	Inpatient	Australia	Yes	Individual	Cardiovascular	None
78	Dyrkorn et al, 2016	C	Outpatient	Europe	Yes	Unknown	Antibiotics	None
79	Elnaem et al, 2019	C	Multiple	Asia	Yes	Both	Cardiovascular	None
80	Fox et al, 2008	NC	Outpatient	N America	Yes	Group	Chronic	IM EHR PF
81	Gadzhanova et al, 2013	NC	Outpatient	Australia	Unknown	Individual	Cardiovascular	AF
82	Graham et al, 2008	C	Outpatient	N America	Yes	Individual	Opioids	None
83	Grover et al, 2013	NC	Outpatient	N America	Yes	Both	Antibiotics	PE
84	Hope et al, 2010	NC	Inpatient	N America	Yes	Group	Chronic	None

© 2025 Rome BN et al. *JAMA Network Open.*

Ref #	Source	Design [a]	Setting	Geographic region	Delivered by clinician	Method	Therapeutic target [c]	Cointerventions [d]
85	Horn et al, 2007	NC	Outpatient	Australia	Unknown	Unknown	Cardiovascular	PE
86	Hughes et al, 2022	NC	Outpatient	N America	Yes	Individual	Opioids	PF
87	Ioannidis et al, 2009	NC	Outpatient	N America	Yes	Group	Chronic	AF PE
88	Jaglal et al, 2009	NC	Outpatient	N America	Yes	Individual	Other	IM PE FU
89	Kattan et al, 2016	C	Outpatient	N America	No	Individual	Opioids	None
90	Keith et al, 2013	NC	Outpatient	Europe	Yes	Group	Polypharmacy	AF
91	Kisuule et al, 2008	NC	Inpatient	N America	Yes	Individual	Antibiotics	None
92	Kiyatkin et al, 2011	NC	Inpatient	N America	Yes	Individual	Antibiotics	None
93	Lee et al, 2021	NC	Inpatient	N America	Unknown	Unknown	Antibiotics	FU
94	Linnebur et al, 2011	C	Nursing home	N America	Yes	Both	Antibiotics	None
95	Liu et al, 2016	NC	Outpatient	Australia	Yes	Individual	Cardiovascular	AF
96	Madaras-Kelly et al, 2022	C	Outpatient	N America	Yes	Individual	Antibiotics	AF IM PF
97	Malmgren et al, 2019	NC	Inpatient	Europe	Yes	Group	Antibiotics	AF EHR
98	Mandryk et al, 2008	NC	Outpatient	Australia	No	Individual	Cardiovascular	None
99	McIntosh et al, 2011	NC	Emergency	Australia	No	Individual	Antibiotics	AF EHR
100	McKeirnan et al, 2021	NC	Outpatient	N America	Yes	Unknown	Vaccines	None
101	Mold et al, 2014	NC	Outpatient	N America	Unknown	Unknown	Chronic	AF PF
102	Monette et al, 2013	NC	Nursing home	N America	Yes	Group	Mental health	None
103	Monette et al, 2008	NC	Nursing home	N America	Yes	Individual	Mental health	FU
104	Montaño et al, 2017	NC	Outpatient	N America	Yes	Individual	Mental health	PE
105	Ndefo et al, 2017	NC	Outpatient	N America	Yes	Individual	Antibiotics	None
106	Neels et al, 2020	NC	Outpatient	Australia	Yes	Individual	Antibiotics	None
107	Oliva et al, 2017	NC	Outpatient	N America	Yes	Individual	Opioids	EHR PE PF
108	Patel et al, 2009	NC	Outpatient	Europe	Yes	Unknown	Mental health	None
109	Paton et al, 2008	NC	Outpatient	Europe	Yes	Individual	Mental health	None
110	Plachouras et al, 2014	C	Outpatient	Europe	Unknown	Unknown	Antibiotics	PE
111	Portman et al, 2022	NC	Outpatient	N America	Yes	individual	antibiotics	AF FU
112	Pratt et al, 2017	NC	Outpatient	Australia	Unknown	Individual	Other	AF PE FU
113	Pruskowski et al, 2021	NC	Nursing home	N America	Yes	Individual	Polypharmacy	None
114	Quanbeck et al, 2018	RCT	Outpatient	N America	Yes	Group	Opioids	AF PF
115	Ragan et al, 2021	NC	Outpatient	N America	Yes	Individual	Mental health	None
116	Reisener et al, 2021	NC	Inpatient	N America	Unknown	Unknown	Opioids	PE PF
117	Roberts et al, 2010	NC	Inpatient	Australia	No	Individual	Interactions	EHR

© 2025 Rome BN et al. *JAMA Network Open.*

Ref #	Source	Design [a]	Setting	Geographic region	Delivered by clinician	Method	Therapeutic target [c]	Cointerventions [d]
118	Saatchi et al, 2021	NC	Outpatient	N America	Unknown	Unknown	Antibiotics	PE
119	Saffore et al, 2020	NC	Outpatient	N America	Yes	Individual	Opioids	None
120	Saffore et al, 2021	C	Outpatient	N America	Yes	Individual	Mental health	None
121	Sanchez et al, 2021	C	Nursing home	Europe	Yes	Group	Polypharmacy	None
122	Smeets et al, 2009	C	Outpatient	Europe	Yes	Both	Antibiotics	AF PE
123	Stafford et al, 2010	NC	Outpatient	N America	Yes	Group	Cardiovascular	None
124	Taylor et al, 2021	C	Outpatient	N America	Yes	Both	Antibiotics	AF
125	Trombetta et al, 2019	C	Outpatient	N America	Yes	Both	Chronic	None
126	Vandenberg et al, 2018	NC	Outpatient	N America	Yes	Group	Polypharmacy	AF EHR
127	Velligan et al, 2021	C	Outpatient	N America	Yes	Group	Mental health	AF PE PF
128	Vinnard et al, 2013	C	Outpatient	N America	Yes	Individual	Antibiotics	None
129	Voelker et al, 2018	NC	Inpatient	N America	Unknown	Unknown	Opioids	None
130	Wang et al, 2021	NC	Outpatient	N America	Yes	Individual	Opioids	AF EHR
131	Wensing et al, 2009	C	Outpatient	Europe	Yes	Group	Multiple	AF
132	Wessell et al, 2008	NC	Outpatient	N America	Yes	Individual	Polypharmacy	AF PF
133	Wood et al, 2022	NC	Inpatient	Australia	yes	group	Opioids	AF PE
134	Wu et al, 2018	NC	Outpatient	Australia	Yes	Both	Antibiotics	None
135	Zgierska et al, 2020	C	Outpatient	N America	Yes	Group	Opioids	PF

[a] RCT = Randomized controlled trial, C = Nonrandomized study with external control, NC = Nonrandomized study with no control

[b] Inpt = Inpatient, Outpt = Outpatient, ED = Emergency department, NH = nursing home

[c] Chronic = other chronic diseases (eg, diabetes, kidney disease, osteoporosis), Polypharmacy = polypharmacy or deprescribing, Interactions = Drug interactions or renal dosing

[d] AF = Audit and feedback, IM = Interactive educational modules, EHR = EHR decision support, PE = patient education, PF = practice facilitation, FU = mailings or other follow-up

© 2025 Rome BN et al. *JAMA Network Open.*
