# Supplementary material for: Academic Detailing Interventions and Evidence-Based Prescribing: A Systematic Review
Source: JAMA Netw Open. 2025 Jan 8;8(1):e2453684. doi: 10.1001/jamanetworkopen.2024.53684 (PMC12543401; doi:10.1001/jamanetworkopen.2024.53684)
Supplement: Supplement 2. — Data Sharing Statement [file jamanetwopen-e2453684-s002.pdf]

Data Sharing Statement

Rome. Academic Detailing Interventions and Evidence-Based Prescribing. *JAMA Netw Open*. Published January 08, 2025. doi:10.1001/jamanetworkopen.2024.53684

Data
Data available: Yes
Data types: Other (please specify)
Additional Information: Full list of articles included in the supplement.
How to access data: Full list of articles included in the supplement.
When available: With publication

Supporting Documents
Document types: None

Additional Information
Who can access the data: Anyone accessing the article.
Types of analyses: Any use.
Mechanisms of data availability: In the supplement.
